# Bacteria-driven hypoxia targeting delivery of chemotherapeutic drug proving outcome of breast cancer

**DOI:** 10.1186/s12951-022-01373-1

**Published:** 2022-04-02

**Authors:** Susu Xiao, Huan Shi, Yan Zhang, Yu Fan, Li Wang, Li Xiang, Yanlin Liu, Ling Zhao, Shaozhi Fu

**Affiliations:** 1 Department of Oncology, The Affiliated Hospital of Southwest Medical University, Luzhou, 646000 People’s Republic of China; 2 Department of Oncology, The Affiliated TCM Hospital of Southwest Medical University, Luzhou, 646000 China; 3 Nuclear Medicine and Molecular Imaging Key Laboratory of Sichuan Province, Luzhou, 646000 China; 4 Department of Pharmaceutics, School of Pharmacy of Southwest Medical University, Luzhou, 646000 China

**Keywords:** Biohybrid, Bifidobacterium infantis, Tumor hypoxia, Albumin nanoparticles, Breast cancer

## Abstract

Local hypoxia is a common feature of many solid tumors and may lead to unsatisfactory chemotherapy outcomes. Anaerobic bacteria that have an affinity to hypoxic areas can be used to achieve targeted drug delivery in tumor tissues. In this study, we developed a biocompatible bacteria/nanoparticles biohybrid (Bif@DOX-NPs) platform that employs the anaerobic *Bifidobacterium infantis* (Bif) to deliver adriamycin-loaded bovine serum albumin nanoparticles (DOX-NPs) into breast tumors. The Bif@DOX-NPs retained the targeting ability of *B. infantis* to hypoxic regions, as well as the cytotoxicity of DOX. The biohybrids were able to actively colonize the hypoxic tumors and significantly increased drug accumulation at the tumor site. The DOX concentration in the tumor masses colonized by Bif@DOX-NPs was 4 times higher than that in the free DOX-treated tumors, which significantly prolonged the median survival of the tumor-bearing mice to 69 days and reduced the toxic side-effects of DOX. Thus, anaerobic bacteria-based biohybrids are a highly promising tool for the targeted treatment of solid tumors with inaccessible hypoxic regions.

**Graphical Abstract:**

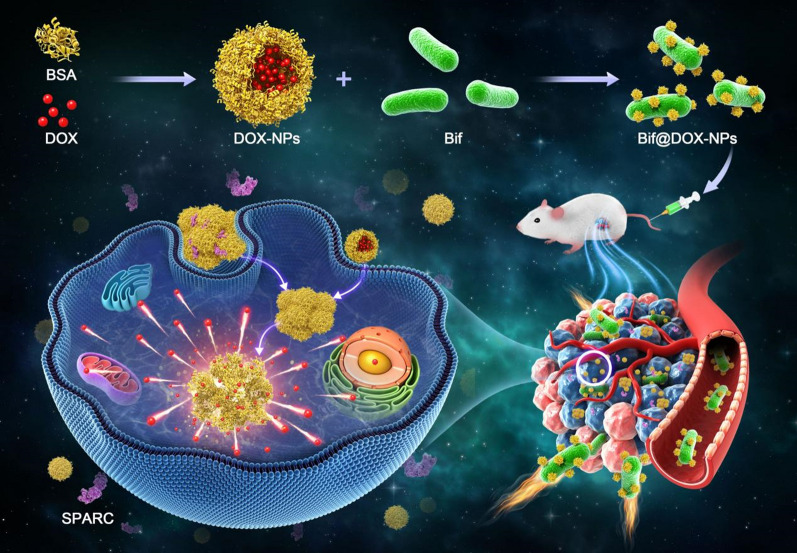

**Supplementary Information:**

The online version contains supplementary material available at 10.1186/s12951-022-01373-1.

## Introduction

Solid tumors are often characterized by central necrosis and chronic hypoxia due to an underdeveloped vascular system and rapid proliferation of tumor cells [1, 2]. The hypoxic environment is a major obstacle to various treatments. Nevertheless, some anaerobic bacteria can selectively colonize the deeper hypoxic regions of solid tumors, and can potentially be used as carriers for chemotherapeutic drugs [3–5]. Several anaerobic bacteria have demonstrated therapeutic effects against inflammatory diseases, metabolic disorders and even cancer, and *Salmonella, Bifidobacterium, Escherichia coli* and *Clostridium perfringens* can colonize solid tumors and directly kill tumor cells [6–10].

**Scheme 1:**
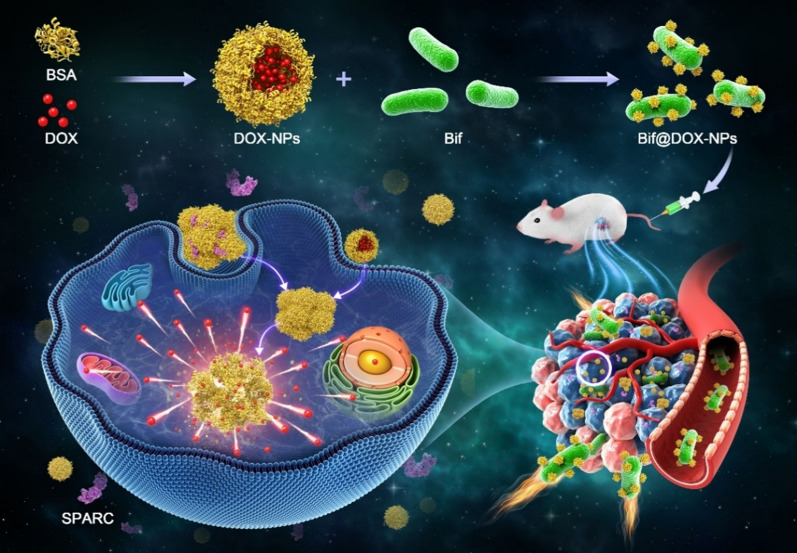
Schematic diagram on the synthesis of bacterial nanohybrids and their treatment of tumors

Nanoparticles (NPs) are increasingly being developed as drug delivery systems since they can improve the water-solubility of hydrophobic drugs and prolong their circulation [11, 12]. However, due to the lack of active targeting ability, most drug-loaded NPs mainly rely on the enhanced permeability and retention (EPR) effect to access tumor tissues [13]. In contrast, incorporation of live anaerobic bacteria on micro/nanoparticles can allow targeted delivery of the drug to hypoxic tumors, resulting in enhanced drug uptake by the tumor cells and lower off-target effects, which translates to synergistically higher anti-tumor effect [14, 15]. The widespread application of attenuated pathogens such as *Salmonella* raises concerns regarding potential immunogenicity that may lead to autoimmune reactions in the host following drug administration [16]. *Bifidobacterium infantis* (Bif) on the other hand is a relatively safe carrier due to its good biocompatibility [17, 18], and can also target tumor-deficient regions, thus allowing drug delivery to tumor tissue [19, 20]. Therefore, we harnessed the tumor-targeting ability of Bif to deliver adriamycin NPs (DOX-NPs) to hypoxic solid tumors in order to improve treatment outcome. Given the tendency of bacterial cells to forage for proteins [46, 47], we coated the DOX with a layer of bovine serum albumin (BSA) and incubated the DOX-NPs with Bif suspension to obtain the Bif@DOX-NPs biohybrids. The preparation process and in vivo performance of the Bif@DOX-NPs was illustrated in Scheme 1. The bacterial hybrids selectively accumulated in the tumors due to the hypoxic targeting ability of Bif, as well as the affinity of the BSA layer to the albumin-binding secreted protein acidic and rich in cysteine (SPARC) [21] that is expressed on many solid tumors [22, 23]. In addition, BSA is non-immunogenic and non-toxic to experimental animals [24, 25].

Bif@DOX-NPs is a promising new tool for targeted anti-cancer therapy that can deliver DOX to the tumor site and decrease its absorption by other organs, thereby reducing the toxic side effects of DOX and improving therapeutic efficacy.

## Results

### Synthesis and characterization of DOX-NPs and Bif@DOX-NPs

**Fig. 1:**
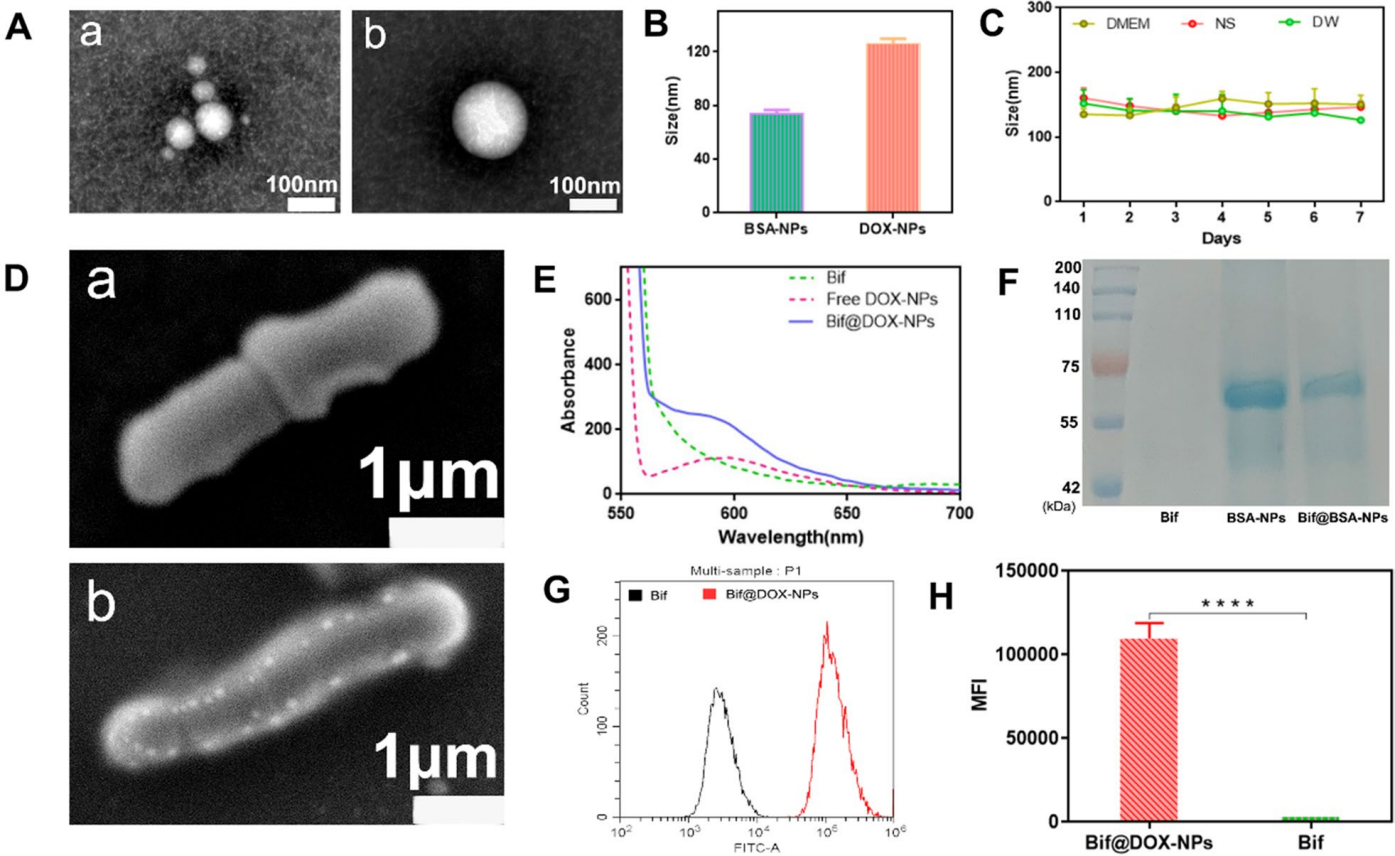
Characterization of DOX-NPs and Bif@DOX-NPs. **A** Morphological features of BSA-NPs (uncoated albumin nanoparticles) and DOX-NPs (albumin nanoparticles encapsulated with adriamycin) captured by TEM. Scale bar, 100 nm. *a: BSA-NPs; b: DOX-NPs.* **B **Average particle size of BSA-NPs and DOX-NPs (n = 3). **C** Particle size stability of DOX-NPs in DMEM, NS and DW. *DMEM* Dulbecco's modified eagle medium,* NS* sanitary saline,* DW* double-distilled water. **D** SEM images of naked Bif (a) and Bif@DOX-NPs (b). Scale bar, 1 μm. **E** Fluorescence spectroscopy analysis of Bif, DOX-NPs and Bif@DOX-NPs. **F** SDS-PAGE protein analysis of Bif, BSA-NPs and Bif@BSA-NPs, samples were stained with Coomassie Brilliant. **G** Flow cytometry analysis of Bif and Bif@DOX-NPs. **H** The relative mean fluorescence intensity (MFI) measured by flow cytometry analysis (n = 3). Asterisks indicate significant differences (****P < 0.0001)

**Fig. 2:**
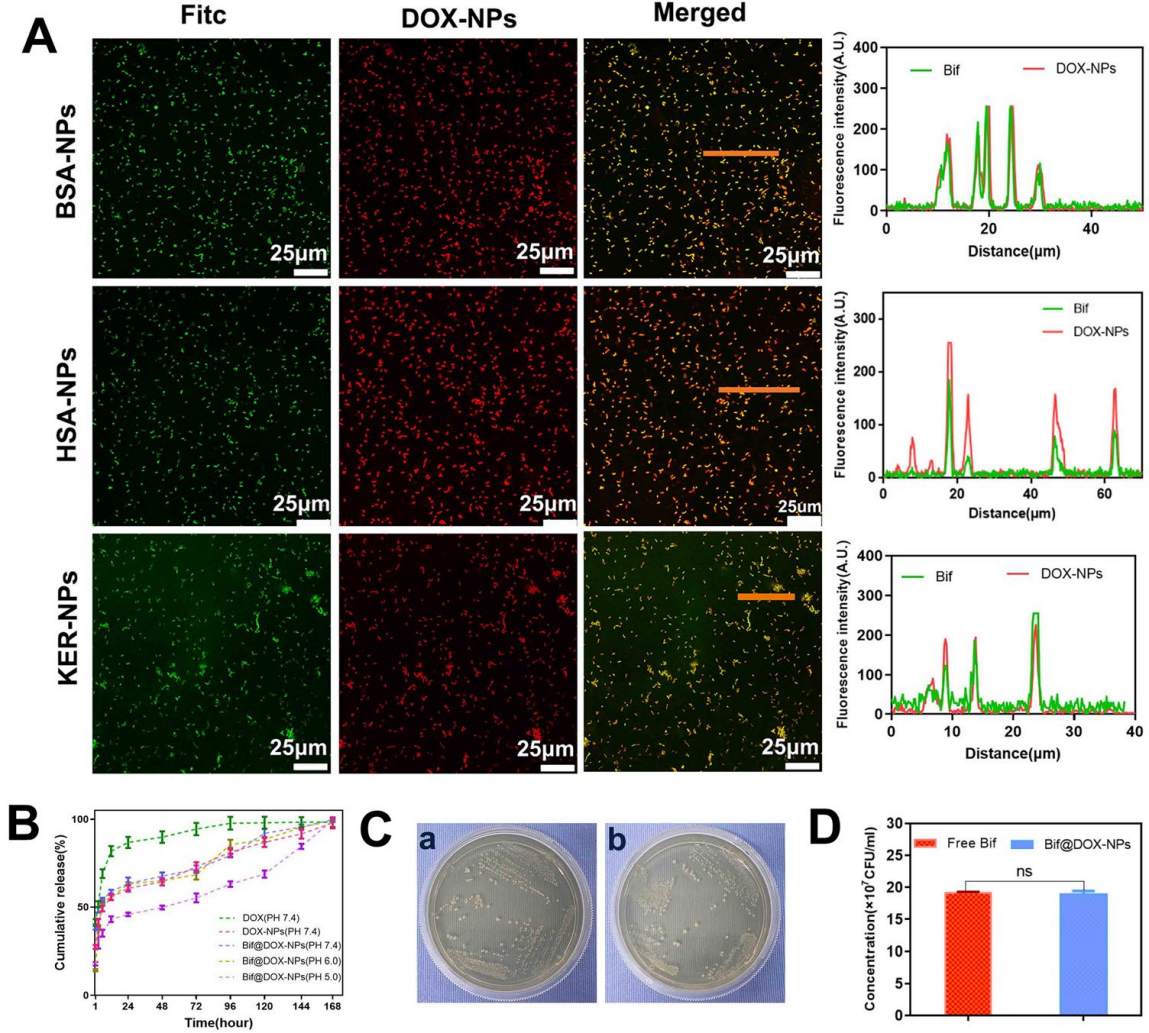
Characterization of Bif@DOX-NPs (*Bifidobacterium infantis* and adriamycin nanoparticle-bound hybrids). **A** CLSM image analysis of the linkage of FITC-labelled Bif and adriamycin nanoparticles prepared from three different nanomaterials (BSA, human serum albumin and keratin). The adriamycin nanoparticles synthesized from the three materials are indicated by BSA-NPs, HSA-NPs, and KER-NPs respectively. The rightmost graph shows the fluorescence co-localization analysis of the three bacterial nanohybrids (Bif@BSA-NPs, Bif@HSA-NPs, Bif@KER-NPs). **B** In vitro release profile of DOX, DOX-NPs in PBS at pH 7.4 and Bif@DOX-NPs at pH 5.0, 6.0 and 7.4 (n = 3, mean ± SD). **C** Anaerobic incubation of Bif and Bif@DOX-NPs for 24 h and counting the number of bacteria in (**D**). Data are presented as mean ± SD (n = 3). (ns: no statistical significance)

The DOX-NPs and BSA-NPs were spherical (Fig. 1A), and the average particle size of DOX-NPs (126.3 nm) was larger than that of the BSA-NPs (74 nm) (Fig. 1B). The DOX-NPs were stable in saline, high sugar medium and double-distilled water in vitro, as indicated by the lack of any significant fluctuations in particle size over a period of one week (Fig. 1C). In addition, Bif, DOX-NPs and Bif@DOX-NPs showed a negative charge potential (Additional file 1: Fig. S1). The drug loading and encapsulation rate of the DOX-NPs were 6.97% and 76.7% respectively. Unlike the smooth surface of naked Bif (Fig. 1Da), the Bif@DOX-NPs consisted of DOX-NPs adhering to the surface of Bif (Fig. 1Db). Furthermore, the fluorescence absorbance of Bif@DOX-NPs was consistent with the pattern of free DOX-NPs (Fig. 1E), indicating successful attachment of DOX-NPs to the surface of Bif. The binding rate of DOX-NPs on Bif was approximately 77.31%. In addition, the protein composition of Bif@BSA-NPs and the BSA-NPs were similar (Fig. 1F). Binding of the DOX-NPs to Bif was also accompanied by increased fluorescence intensity of the Bif@DOX-NPs (Fig. 1G, H). These results all confirmed the successful construction of the Bif@DOX-NPs biohybrids. The characteristic binding between Bif and protein-based NPs was also verified using HAS-NPs and KER-NPs through fluorescence co-localization (Fig. 2A). As shown in Fig. 2B, the cumulative amounts of DOX released from the Bif@DOX-NPs and DOX-NPs over a 12 h period were 55.54 ± 1.58% and 58.8 ± 1.04% respectively, while 81.84 ± 2.95% of the free DOX was released within the same time period. This suggested that the binding of DOX-NPs onto Bif did not affect the slow-release pattern of DOX-NPs. In addition, the DOX release rate from Bif@DOX-NPs was unaffected at near neutral pH of 6.0 and 7.4, but slowed to 43.12 ± 1.63% in 12 h at pH 5.0. Finally, the binding of DOX-NPs onto Bif did not affect bacterial growth, as indicated by the similar number of colonies formed by Bif and Bif@DOX-NPs in 24 h (P > 0.05; Fig. 2C, D). As shown in Figure S2AC, no significant dissociation occurred in PBS (pH = 7.4) containing Bif@DOX-NPs at 0 h and 24 h, while an obvious change in the color of the supernatant was clearly observed when MMP-2 was added (Additional file 1: Fig. S2B). The dissociation rate of DOX-NPs significantly increased from 35.78 ± 0.09% (Control) to 62.06 ± 0.06% after addition of MMP-2 (P < 0.0001, Additional file 1: Fig. S2D), indicating that DOX-NPs can fall off from the Bif@DOX-NPs biohybrids owing to the digestion of MMP-2.

### In vitro cellular experiments

**Fig. 3:**
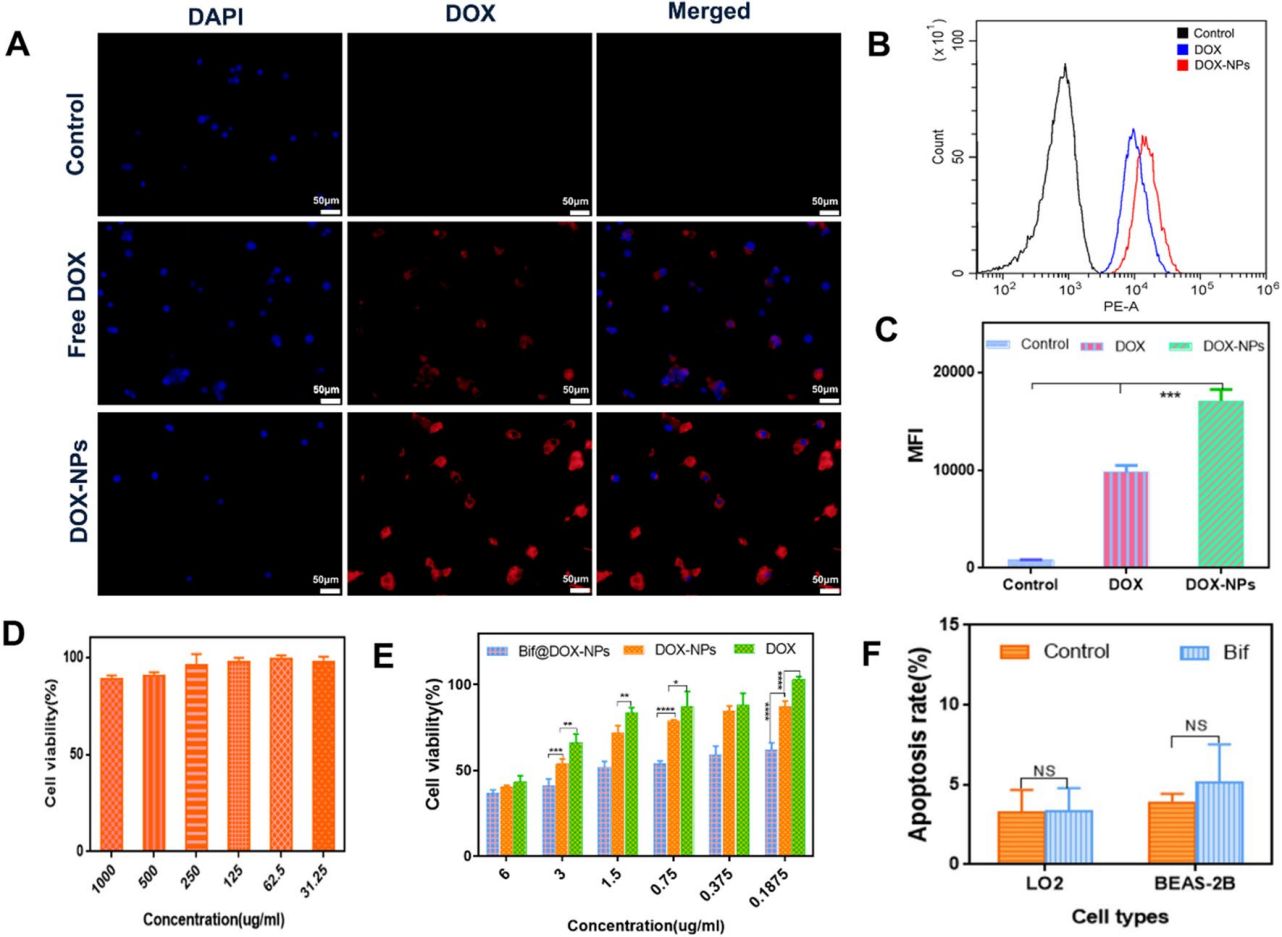
Cellular uptake and cytotoxicity. **A** The fluorescence microscopy microscopic images of DOX and DOX-NPs uptake by 4T1 cells. **B** Flow cytometry analysis of DOX and DOX-NPs uptake by 4T1 cells and **C** The relative mean fluorescence intensity (MFI) corresponding to flow cytometry analysis. Data are presented as mean ± SD (n = 3, ***P < 0.001). **D** The cytotoxicity of BSA-NPs on 4T1 cells (n = 6). **E** The cytotoxicity of DOX, DOX-NPs and Bif@DOX-NPs on 4T1 cells. Data are presented as mean ± SD (n = 3, *P < 0.05, **P < 0.01, ***P < 0.001, ****P < 0.0001). **F** Flow cytometry analysis of apoptosis rates of liver cells (LO2) and lung cells (BEAS-2B) induced by Bif. Data are presented as mean ± SD (n = 3, *ns* no statistical significance)

**Fig. 4:**
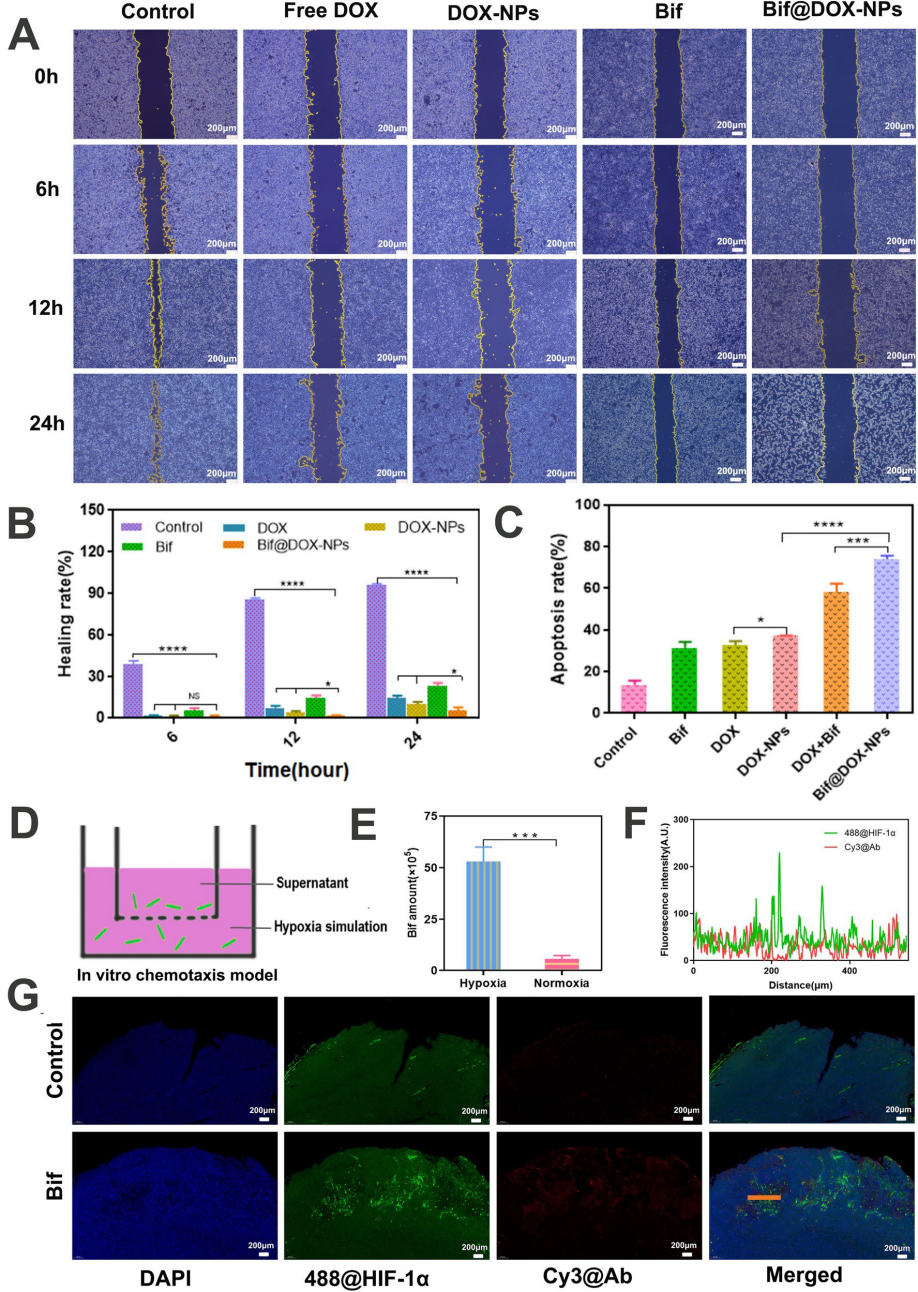
In vitro invasiveness and cytotoxicity of various treatments on 4T1 cells. **A** Wound healing. **B** The healing rate of Control, DOX, DOX-NPs, Bif and Bif@DOX-NPs at 6, 12 and 24 h. **C** Flow cytometry analysis of apoptosis rates of 4T1 cells induced by different preparation groups. **D** Schematic illustration of the hypoxia simulation model using a Transwell system to evaluate the chemotaxis of Bif@DOX-NPs. **E** Number of bacteria migrating to the bottom chamber. **F** Bif@DOX-NPs and hypoxic zone co-localization in vivo. Hypoxic zone was stained green with 488@HIF-1α (Anaerobic induction factors labelled with Alexa Fluor 488), and Bif was stained red with Cy3@Ab (*Bifidobacterium infantis* antibodies labelled with Cy3). (G) Fluorescence co-localization analysis of Bif@DOX-NPs and hypoxic zone. Results are expressed as mean ± SD (n = 3, *ns* no statistical significance, *P < 0.05, ***P < 0.001, ****P < 0.0001)

As shown in Fig. 3A, the DOX-NPs were more readily taken up by 4T1 cells compared to free DOX, which was also quantified in terms of the fluorescence intensity (P < 0.001; Fig. 3B, C). The lower uptake of free DOX translated to significantly weaker inhibitory effect on the tumor cells compared to the DOX-NPs, Bif and Bif@DOX-NPs, with the latter exhibiting the strongest cell-killing activity (Fig. 3E). In contrast, the BSA-NPs were not cytotoxic (Fig. 3D). Furthermore, Bif did not have any significant toxic effects on the normal hepatocytes (LO2) and lung cells (BEAS-2B) (Fig. 3F, Additional file 1: Fig. S3). In vitro wound healing assay indicated that the Bif@DOX-NPs significantly inhibited tumor cell migration compared to the free bacteria and other drugs. As shown in Fig. 4A, the wound region of the cellular monolayer treated with Bif@DOX-NPs was practically “unhealed”, with the least migration rate of 5.33% after 24 h compared to the other groups (P < 0.05, Fig. 4B). Consistent with this, the apoptosis rate in the Bif@DOX-NPs group was the highest amongst all groups at 72.47% (P < 0.001, Fig. 4C, Additional file 1: Fig. S4). The apoptosis rate of the Bif-treated 4T1 cells was 2.3 times higher than that of the untreated controls. In addition, the apoptosis rates of the hepatocellular carcinoma Huh7 and HepG2 cell lines treated with Bif were 2.04-fold and 1.23-fold higher than that of the respective controls (P < 0.001, Additional file 1: Fig. S5).

### Hypoxic targeting activity of Bif@DOX-NPs

**Fig. 5:**
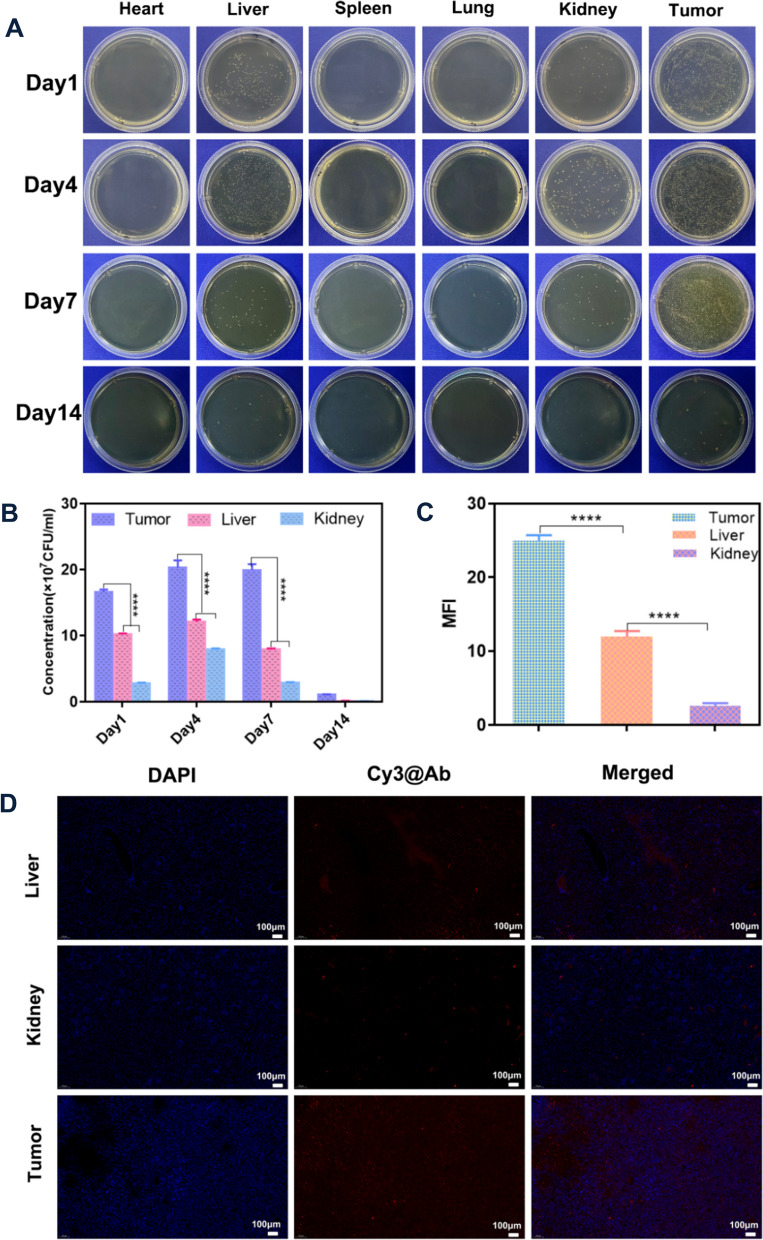
In vivo biodistribution of Bif@DOX-NPs. **A** Homogenates of tumor tissues and the five organs were cultured on agar plates under an anaerobic environment at 37 ℃. **B** Bacterial growth was measured on days 1, 4, 7, and 14. **C** The relative mean fluorescence intensity (MFI) of Cy3@Ab in tumor, liver and lung. **D** Indirect observation of bifidobacterial localization in tumors, liver and lungs with Cy3@Ab. Data are presented as mean ± SD (n = 3, ****P < 0.0001)

To evaluate the hypoxic targeting ability of the bacterial hybrids, we simulated an anaerobic environment in vitro using Transwell chambers (Fig. 4D). As shown in Fig. 4E, a significant proportion of the bacteria inoculated into the upper normoxic chamber migrated to the bottom hypoxic chamber (P < 0.001). Consistent with this, the tumor tissues from mice injected with Bif showed clear localization of the (Cy3-labeled) bacteria in the hypoxic zone (Alexa Fluor 488-labeled HIF-1α) of the tumors (Fig. 4F, G). In addition, Bif was mainly distributed in the liver, kidney and tumor tissues on day 1 and day 4 after inoculation. On day 7, there was a significant decrease in the bacterial load of liver and kidney, whereas the tumor tissues were completely colonized (Fig. 5A). After two weeks, the bacteria were largely cleared from all tissues (Fig. 5B, C). In contrast, no bacteria could be found in tumor tissues in the NS group (Additional file 1: Fig. S6). Fluorescence co-localization of bacteria in liver, kidney and tumor tissues also confirmed the preferential colonization of the hypoxic tumors (P < 0.0001; Fig. 5D).

### In vivo anti-tumor efficacy

**Fig. 6:**
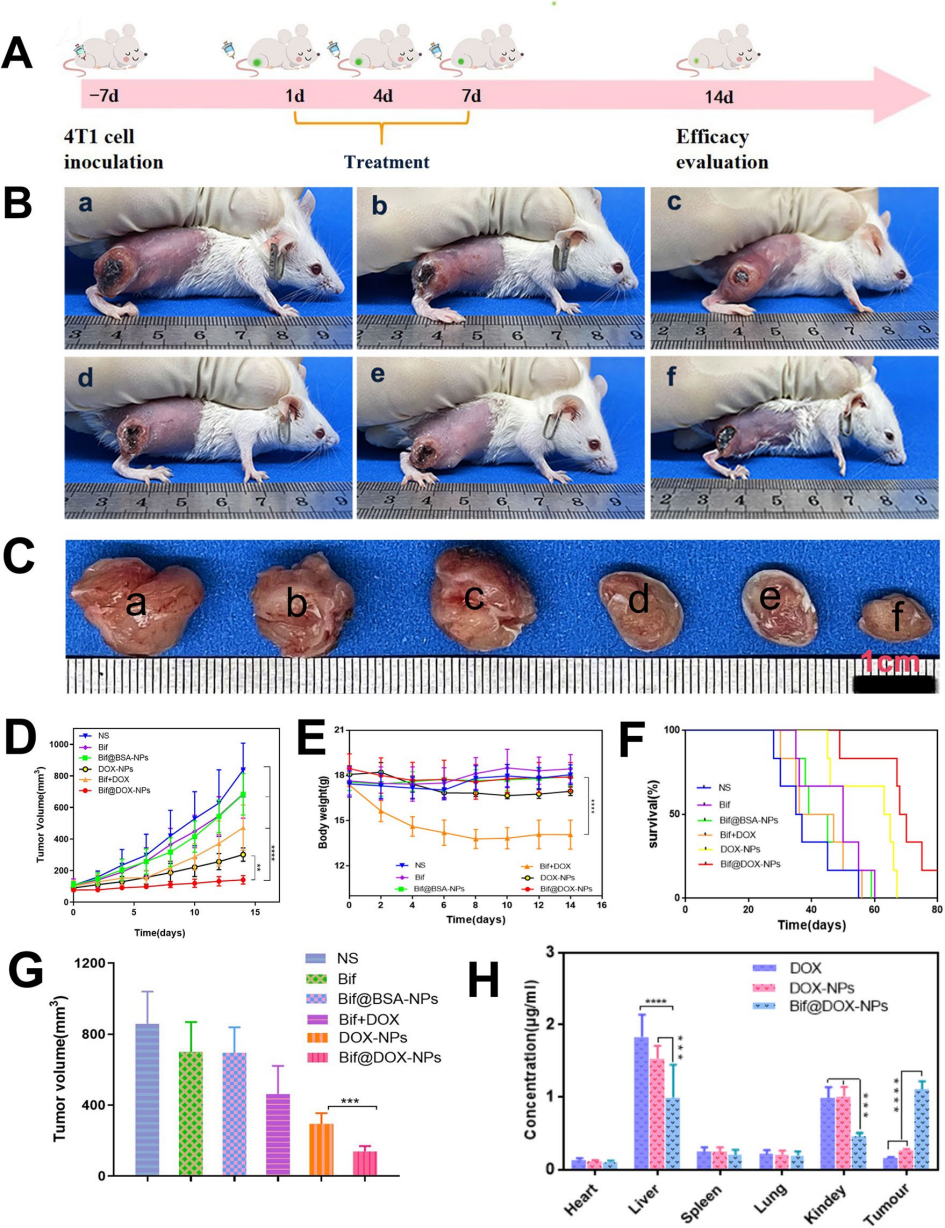
Anti-tumor activity of Bif@DOX-NPs against 4T1 tumors in mice. Tumor growth curves (**D**), body weight curves (**E**) and survival curves (**F**) of mice receiving different therapeutic regimens as shown in panel **A** (n = 6, **P < 0.01, ****P < 0.0001). **B** Representative photos of 4T1 tumor-bearing mice on the 14th day after various treatments*. a: Control, b: Bif, c: Bif@BSA-NPs, d: DOX* + *Bif, e: DOX-NPs, f: Bif@DOX-NPs*. **C** Representative photographs of isolated tumors. a to f means the same group as above. **G** The tumor volume of mice during different treatments at 14 d. **H** In vivo drug concentration distribution. Results are expressed as mean ± SD (n = 3, ***P < 0.001, ****P < 0.0001)

**Fig. 7:**
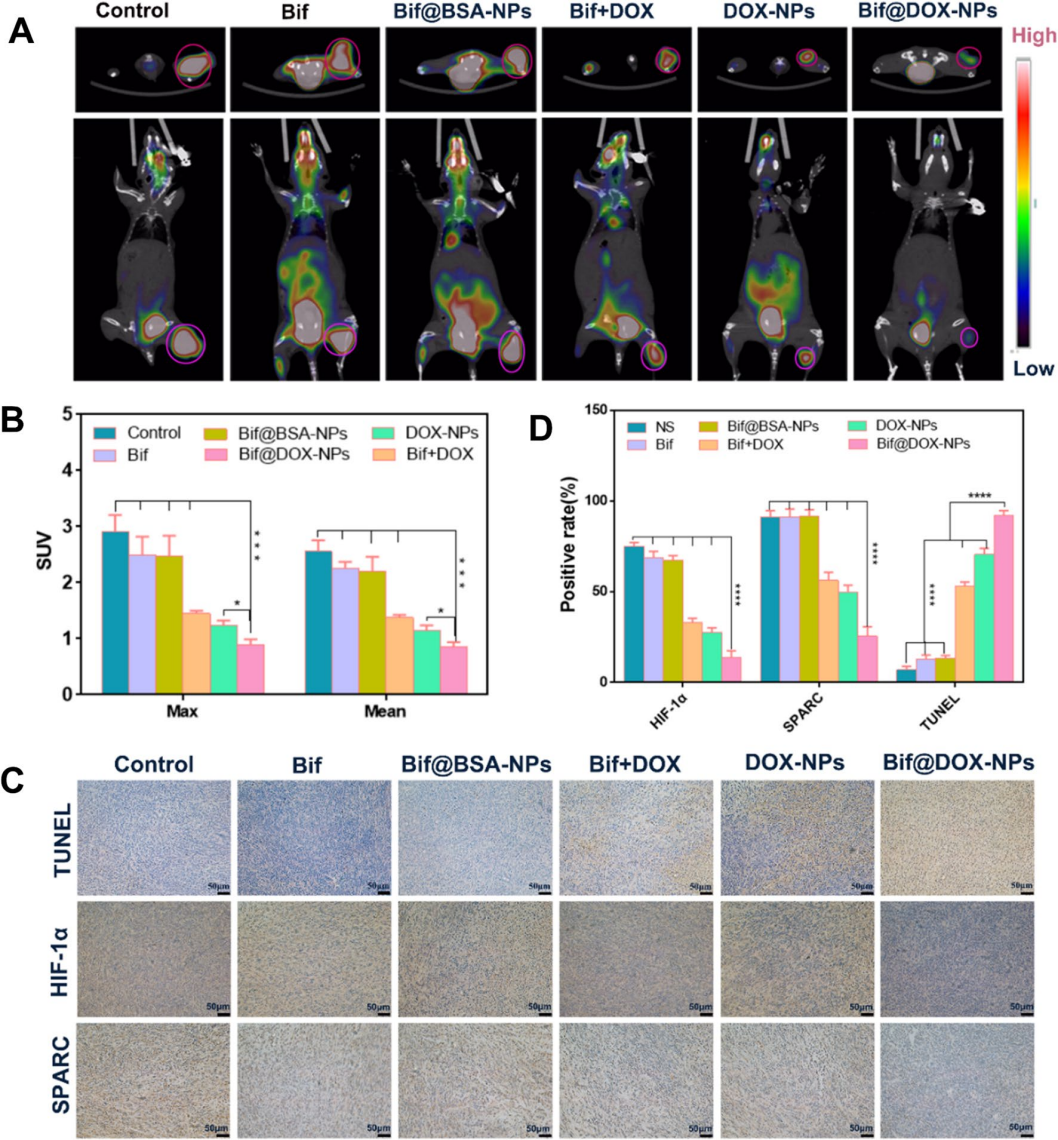
Micro-PET/CT imaging and immunohistochemical analysis of tumor tissues after various treatments. **A** The images of 18F-FDG PET/CT scanning in mice on the 14th day after various treatments. **B** SUVmax and SUVmean values for various groups. **C** Representative micrographs of tumor tissue stained by TUNEL and immunohistochemistry to detect cleaved HIF-1α and SPARC in tumors. Scale bar, 50 μm. **D** The positive expression rates of TUNEL, HIF-1α and SPARC in tumor. Results are expressed as mean ± SD (n = 3). Asterisks indicate significant differences (ns: no statistical significance, *P < 0.05, ***P < 0.001, ****P < 0.0001)

The treatment regimen is outlined in Fig. 6A. The mice treated with the Bif@DOX-NPs had the smallest tumors and the slowest tumor growth rate compared to the other groups (P < 0.01; Fig. 6B-D, G). The body weight of the tumor-bearing mice fluctuated slightly except for the Bif + DOX group that showed a significant decrease (Fig. 6E), indicating that free DOX had a stronger toxic effect. Consistent with this, the median survival duration of mice in the NS, Bif, Bif@BSA-NPs, Bif + DOX and DOX-NPs groups was 36, 50, 42, 41.5 and 64 days respectively, compared to 69 days in the Bif@DOX-NPs group (Fig. 6F). As shown in Fig. 6H, the intra-tumoral DOX concentration in the Bif@DOX-NPs group was higher than that in mice treated with free DOX or DOX-NPs, which corresponded to lower DOX accumulation in the liver and kidney of the Bif@DOX-NPs group (P < 0.001). Furthermore, 18F-FDG micro-PET/CT scanning showed the least tumor uptake of FDG in the Bif@DOX-NPs group compared to the other groups (Fig. 7A), indicating that Bif@DOX-NPs significantly inhibited tumor growth and metabolism. The SUV_max_ in the Bif@DOX-NPs group was only 1.03 ± 0.052 compared to 2.9 ± 0.3, 2.597 ± 0.327, 2.36 ± 0.3, 1.443 ± 0.051 and 1.23 ± 0.089 in the NS, Bif, Bif@BSA-NPs, Bif + DOX and DOX-NPs groups respectively (p < 0.05, Fig. 7B). The SUV_mean_ values also presented a similar trend (Fig. 7B). The lowest uptake of 18FDG in the Bif@DOX-NPs group was indicative of the best early treatment response.

TUNEL staining of tumor tissues showed a higher density of apoptotic cells in the Bif@DOX-NPs group compared to the other groups (Fig. 7C). The percentage of apoptotic cells in the NS, Bif, Bif@BSA-NPs, Bif + DOX and DOX-NPs groups were respectively 7 ± 2%, 12.66 ± 2.51%, 13.33 ± 1.52%, 2.66 ± 2.51% and 70.33 ± 3.51% compared to 92 ± 2.64% in the Bif@DOX-NPs group (P < 0.0001; Fig. 7D), indicating that the latter promoted apoptosis of tumor cells. The in situ expression of HIF-1α in the Bif@DOX-NPs group was 14 ± 2.6%, which was significantly lower compared to the other groups (P < 0.0001; Fig. 7C, D), indicating that Bif@DOX-NPs alleviated hypoxia in tumors. Similarly, the percentage of SPARC positive cells in the Bif@DOX-NPs group was also markedly lower compared to that in the other groups at 25.66 ± 5.13% (P < 0.0001; Fig. 7D).

### In vitro hemolysis and in vivo toxicity of Bif@DOX-NPs biohybrids

**Fig. 8:**
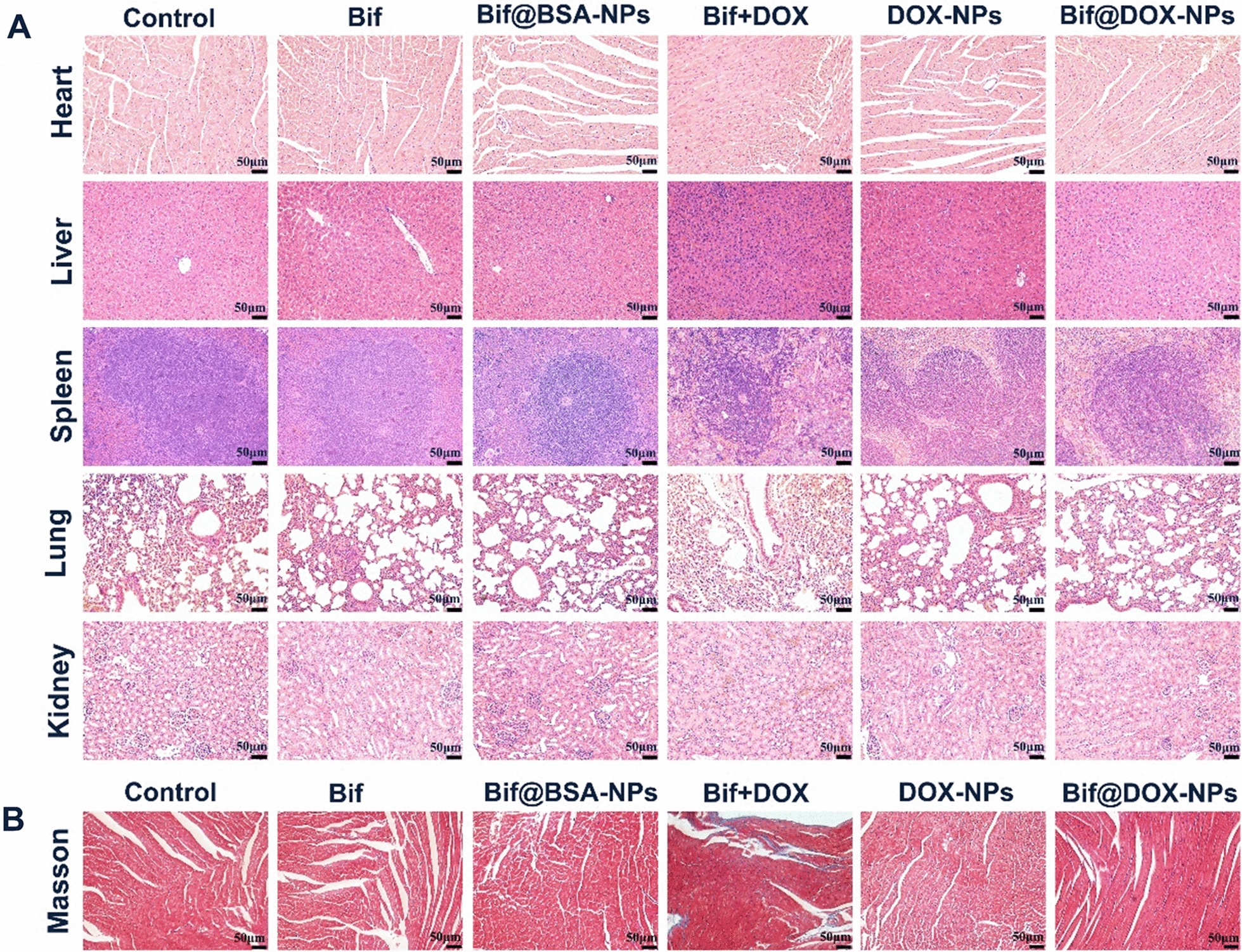
In vivo biocompatibility evaluation. **A** Hematoxylin and eosin staining of tumor tissues and major organs (including heart, liver, spleen, lung and kidney) after treatment as indicated. Scale bar, 50 μm. **B** Masson staining of heart after treatment as indicated. Scale bar, 50 μm

As shown in Additional file 1: Fig. S7A, B, double-distilled water caused complete erythrocyte rupture (100% hemolysis), whereas no significant hemolysis was observed in saline and other solutions. The UV–vis absorbance spectroscopy (Additional file 1: Fig. S7C) and hemoglobin concentration (Additional file 1: Fig. S7D) in each group also verified these results. Compared to the complete (100%) hemolysis observed in double-distilled water, the hemolysis rates in Bif@BSA-NPs, BSA-NPs and Bif solutions were significantly lower at 2.807%, 2.072% and 2.373% respectively. Thus, Bif and BSA-NPs are essentially non-lethal to erythrocytes. Furthermore, HE staining of heart, liver, spleen, lung and kidney showed no significant organ damage in any of the groups (Fig. 8A). Masson staining of the cardiac tissues demonstrated slight myocardial fibrosis only in the free DOX group (Fig. 8B). The Bif@DOX-NPs biohybrids had no significant effect on hematological parameters including WBC, RBC, HGB and PLT, whereas a decrease in leukocyte counts was observed only in the Bif + DOX group. In addition, Bif@DOX-NPs did not induce any significant changes in the biochemical indices of heart, liver and kidney function, and the combination of Bif and DOX resulted in a significant increase in AST, ALT and CK levels, which were indicative of liver and kidney damage (Additional file 1: Fig. S8).

## Discussion

The combination of inadequate blood vessels and the rapid proliferation and metabolism of malignant cells in the deeper regions of solid tumors induces a hypoxic environment [26, 27], which often results in sub-optimal treatment response [28]. Although nano-formulations have now entered clinical applications and exhibit high therapeutic efficacy with minimal side effects, they mainly depend on EPR or active targeting via receptor-ligand interaction for selective accumulation in tumors [29]. Therefore, nano-therapies have limited efficacy due to ineffective targeting and penetration of the NPs [13].

Interestingly, anaerobic bacteria can actively target the hypoxic tumor areas [30], and efficiently deliver therapeutic agents to the deep hypoxic regions of solid tumors. The bacterial cells can be loaded with drugs through chemical bonding [31], charge attraction [32] antigen–antibody binding [33] and biotin-streptavidin interaction [34]. Cai et al*.* generated bacterial/nanomaterial complexes by linking *Salmonella* with NPs via amide bonds [15]. In addition, positively charged NPs can adhere to the surface of invasive *Salmonella* by electrostatic interaction [35]. Behkam et al*.* linked *Salmonella* to NPs through streptavidin–biotin interactions [36], whereas Yeh et al*.* used antigen–antibody binding to guide NPs to hypoxic tumor sites colonized by anaerobic bacteria [7]. However, the aforementioned strategies involve modifying the bacterial cells and/or NPs, or the addition of foreign substances, which may affect bacterial activity or induce other changes in the microorganisms. Moreover, most groups so far have used pathogenic *E. coli* or *Salmonella* to load NPs, which require attenuation prior to use. [37, 38] In our study, we harnessed the tendency of bacterial cells to forage for protein-like substances to directly attach albumin-coated DOX-NPs onto the surface of Bif cells. This obviated the need for bacterial attenuation or modification, or a catalyst to aid the binding process. Therefore, the Bif@DOX-NPs biohybrids retained their biological activities, and were also non-toxic in vivo. Moreover, DOX-NPs can stably bind to Bif, whereas in the tumor microenvironment DOX-NPs are more likely to fall off, which may result from the over-expression of matrix metalloproteinase-2 (MMP-2) in tumor tissues [48, 49]. The spontaneous tropism of Bif for proteins was further confirmed by the formation of biohybrids with other protein-based NPs (HAS-NPs and KER-NPs).

*B. infantis* can not only target hypoxic regions of solid tumors [39, 40] but is also a safe probiotic for humans [17, 18]. The Bif did not induce hemolysis or apoptosis in normal hepatopulmonary cells, and had no obvious effect on the heart, liver, spleen, lung and kidney function in mice. Thus, Bif is a highly promising carrier for targeted delivery of chemotherapeutic drugs to the hypoxic zone of solid tumors. Interestingly, Bif had an inhibitory effect on the growth of tumor cells and even promoted apoptosis. This is consistent with previous studies [19, 40] that have reported that probiotic microorganisms such as *Bifidobacteria* can exert anti-cancer effects by producing antioxidant enzymes, scavenging reactive oxygen species, chelating heavy metals and neutralizing different carcinogens. In addition, they can regulate the cell cycle of cancer cells, inhibit their proliferation and sensitize them to apoptosis [41, 42].

Apart from tumor-targeting ability, the Bif@DOX-NPs biohybrids also achieved sustained DOX release, which retained the cytotoxic effects of DOX and also reduced DOX-induced cardiotoxicity [43]. Furthermore, the blank BSA-NPs were non-toxic even at high concentration of 1000 μg/mL. BSA-based nano-formulations have clinical translation potential since protein-based nanodrugs have been used successfully in the clinic [22]. Albumin NPs can bind to gp60 receptors on the surface of tumor vascular endothelial cells, resulting in invagination of the cell membrane and the generation of trans-cellular vesicles, which allows the NPs to cross the endothelium and reach the tumor tissue interstitial space [44]. Tumors secrete cysteine-rich acidic proteins such as SPARC during growth that attract adherent albumin, thereby allowing drug translocation from the tissue interstitium into the tumor cells [45], eventually increasing the intra-tumoral accumulation of albumin NPs [21]. SPARC is overexpressed in many solid tumors [22, 23], and its in-situ expression decreased significantly in the tumor tissues treated with Bif@DOX-NPs, indicating its good anti-tumor efficacy.

Engineered bacteria or cells as drug delivery carriers are playing a more and more important role in the diagnosis and treatment of malignant tumors [50, 51]. This study combines anaerobic bacteria with nanotechnology to construct a bacteria/nanoparticles biohybrids for delivery of chemotherapeutic agent. *B. infantis* can effectively deliver chemotherapy drugs to hypoxic tumor regions, thereby increasing intra-tumoral drug accumulation and reducing extra-tumoral absorption. This can not only improve the therapeutic outcome but also minimize toxic side-effects. Biohybrids of bacteria and nanomedicine are novel tools for the treatment of solid tumors.

## Conclusion

We generated Bif@DOX-NPs biohybrids by binding DOX-NPs on the surface of *Bifidobacterium infantis* for the targeted delivery of chemotherapeutic drugs to hypoxic tumor sites. The Bif@DOX-NPs improved the therapeutic efficacy of DOX by increasing intra-tumoral drug accumulation and inducing tumor cell apoptosis. The biohybrids also reduced drug absorption by normal tissues and decreased the toxic side effects of DOX. Therefore, Bif@DOX-NPs biohybrids might play a promising role in the treatment of solid tumors.

## Materials and methods

### Reagents, animals and bacteria

BSA was purchased from Meilun Biological Technology Co. Ltd. (Dalian, China). Adriamycin hydrochloride was purchased from Macklin Biochemical Co. Ltd. (Shanghai, China). Female Blab/c mice weighting 16–18 g (6 weeks of age) were purchased from Tengxin Biological Technology Co. Ltd. (Chongqing, China). All animal experimental procedures were approved by the Ethics and Science Committee of the Animal Care and Treatment Committee of Southwest Medical University. Balb/c mice were purchased from Tengxin Biotechnology Co. Ltd., Chongqing, China). The mice were housed under specific pathogen-free conditions at 24 °C and relative humidity of 50–60% under a 12-h-light/12-h-dark schedule, with ad libitum access to standard rodent food and tap water. All the mice were healthy and had no infection during the experimental period. *B. infantis* (GIMI.207) was purchased from the Strains Preservation Center of Guangzhou Institute of Microbiology (Guangdong, China), and incubated anaerobically on agar plates at 37 °C for 48 h. The colonies were collected in sterile dry centrifuge tubes and centrifuged at 2000 rpm for 5 min to obtain single cell suspensions.

### Synthesis and characterization of DOX-NPs and biohybrid Bif@DOX-NPs

DOX and BSA were dissolved in double-distilled water and stirred for 2 h at room temperature, and the pH was adjusted to 7 ~ 10. Ethanol was added dropwise at the rate of 0.5 mL/min and the solution was stirred continuously. The components were cross-linked by adding 20 μL 8% glutaraldehyde at room temperature for 12–24 h. The organic solvent was then removed using a rotary evaporator (RE, R201, Shenshun Biotechnology Co., Shanghai, China) and the resulting NPs were harvested. The morphology of the DOX-NPs was observed by transmission electron microscopy (TEM, Tecnai G2 F20, FEI, USA). Particle size and zeta potential were measured using dynamic light scattering (DLS, NanoBrook90 plus Zeta, Brookhaven, NY) at 25 °C. In vitro stability of DOX-NPs in PBS, DMEM and distilled water (DW) was monitored by measuring particle size. Drug loading (DL) and encapsulation efficiency (EE) were determined by UV spectrophotometry (UV-5800PC, Shanghai Metash Instruments Co., Ltd., Shanghai, China) at 25 °C. Briefly, the samples were dissolved in double-distilled water and OD at 485 nm was measured. DL and EE were calculated using the following equations.

$$\mathrm{DL}=\frac{Drug}{(BSA+Drug)}\times 100\mathrm{\%}$$

$$\mathrm{EE}=\frac{Actual \, DL}{Theoretical \, DL}\times 100\mathrm{\%}$$

To construct Bif@DOX-NPs biohybrids, Bif was incubated anaerobically in agar medium at 37 °C for 48 h. The bacterial colonies were resuspended, washed thrice, and the Bif suspension (2.0 × 10^7^ cfu/mL) was incubated anaerobically at 37 °C with DOX-NPs (40ug/mL) for 4 h. The solution was centrifuged at 2000 rpm for 5 min, and the supernatant was removed and washed twice to obtain the Bif@DOX-NPs biohybrids. The surface morphology of Bif@DOX-NPs was imaged with a field emission scanning electron microscope (SEM, Hitachi S3400N, Japan). Bif@HSA-NPs and Bif@KER-NPs were similarly prepared using human serum albumin (HAS) and keratin (KER) instead of BSA. To ascertain the co-localization of Bif and the protein NPs, the bacterial cells were labelled with FITC prior to constructing the biohybrids, which were then observed by confocal laser scanning microscopy (Zeiss LSM 880, Carl Zeiss, Germany). The fluorescence absorption of Bif@DOX-NPs was measured by fluorescence spectrophotometry (Perkin-Elmer LS-55, Smeo Analytical Instruments Co, China). Flow cytometry (BD FACSVerse, Piscataway, NJ) was used to quantify the fluorescence intensity of adriamycin in Bif@DOX-NPs. To determine whether the loading of DOX-NPs on the surface of Bif affected its activity, equal amounts of Bif@DOX-NPs and Bif were inoculated onto agar plates and the colonies were counted after 24 h. The binding stability of Bif@DOX-NPs was also evaluated. The Bif@DOX-NPs were resuspended in PBS (pH = 7.4). The matrix metalloproteinase 2 (MMP-2) was added to the Bif@DOX-NPs solution and incubated for 24 h. The solution without MMP-2 was used as control. Then the solutions were centrifuged and the color change of supernatant was photoed. The fluorescence intensity of the supernatant was measured and used to calculate the dissociation rate of DOX-NPs from Bif@DOX-NPs biohybrids.

### In vitro drug release, cell uptake and cytotoxicity assays

The samples were put into dialysis bags (molecular weight cutoff 3500 Da) that were then placed into 40 mL PBS (pH = 7.4, 6.0, 5.0) containing 1% Tween 80 (v/v) in a water bath shaker (37 ± 0.5 °C). At the stipulated time points, 3 ml media was collected for analysis and replaced with the equal amount of PBS. The amount of the drug in the buffer was measured by UV–Vis spectrophotometry (UV-5800PC, Shanghai Metash Instruments Co., Ltd., Shanghai, China).

To measure cellular uptake of the NPs, 4T1 cells were seeded in 6-well plates at the density of 2 × 10^5^ cells/mL and incubated with normal saline (NS), free DOX or DOX-NPs for 2 h. The cells were then stained with DAPI for 5–10 min, washed thrice with PBS, and imaged by a fluorescence microscope (OLYMPUS, IX73, Japan). The fluorescence intensity of the internalized DOX was quantified by flow cytometry (BD FACSVerse, Piscataway, NJ) [52]. For the cytotoxicity assay, 4T1 cells were seeded into 96-well plates and treated with DOX, DOX-NPs or Bif@DOX-NPs for 24 h, and the MTT solution was then added to each well, after incubating the cells for another 4 h, 100 μL dimethyl sulfoxide was added to each well, and the OD was measured. To investigate the effect of Bif on normal LO_2_ cells and BEAS-2B cells, the two kinds of cells were seeded into 6-well plates (5 × 10^4^/well) and incubated with Bif for 24 h. Then the cells were collected and resuspended by adding Annexin V-mCherry Binding Buffer, then stained with Annexin V-mCherry and SYTOX Green for 10–20 min, finally apoptosis was detected by flow cytometry (BD FACSVerse, Piscataway, NJ).

### In vitro apoptosis and wound healing assays

The 4T1 cells were seeded into 6-well plates (5 × 10^4^/well) and incubated with NS, Bif, DOX, DOX-NPs, Bif + DOX or Bif@DOX-NP for 24 h. The cells were harvested, resuspended in Annexin V-mCherry Binding Buffer and then stained with Annexin V-mCherry and SYTOX Green for 10–20 min. The percentage of apoptotic cells were detected by flow cytometry (BD FACSVerse, Piscataway, NJ). The effect of Bif on the apoptosis rates of normal hepatopulmonary cells (LO2 cells and BEAS-2B cells) as well as hepatocellular carcinoma cell lines was evaluated by the same procedure. For the in vitro wound healing assay, 4T1 cells were cultured till confluent and the monolayer was scratched using a sterile pipette tip. After washing off the dislodged cells, fresh DMEM containing Bif, DOX, DOX-NPs, Bif + DOX or Bif@DOX-NPs was added. The wound coverage area was photographed 6, 12 and 24 h and the migration rate was calculated.

### Evaluation of the anaerobic targeting of bacterial/NPs biohybrids

The top compartment of Transwell insert was filled with Bif@NPs (200 μL, 5 × 10^7^ cfu/mL) and the bottom with 0.4 mL mixture of glucose (0.4 mg/mL), glucose oxidase (0.5 kU) and catalase (0.5 kU). The oxidation of glucose by glucose oxidase depleted the oxygen, and the hydrogen peroxide produced during this reaction was quenched by catalase, resulting in a hypoxic environment. In the control group, the bottom compartment was filled with 0.4 mL glucose solution. After 2 h of incubation, the Bif@NPS in the bottom compartment were harvested and the number of cells was counted. Bif@DOX-NPs were injected into tumor-bearing mice through the tail vein, and tumor tissues were harvested 24 h later. The tissue sections were stained with Cy3-labeled anti-Bif antibody (Cy3@Ab) and Alexa Fluor 488-labeled anaerobic induction factor (488@HIF-1α) to demarcate the hypoxic zone, and the nuclei were counterstained with DAPI. The distribution of Bif in the hypoxic sites was observed under a fluorescence microscope and counted. In another experiment, tumor-bearing mice were injected with Bif@DOX-NPs or NS and euthanized 1-, 4-, 7- and 14 days post-injection. The major organs (heart, liver, spleen, lung and kidney) and tumor tissues were harvested, and homogenized in sterile water containing 0.1% Triton X-100. The tissue homogenates were serially diluted and incubated on solid LB agar plates at 37 °C for 24 h. The colonies were counted and photographed. The organs with high bacterial colonization (liver and kidney) and the tumor tissues were embedded in paraffin, cut into sections, and stained as described above. The fluorescence intensity in each tissue was observed by fluorescence microscopy and statistically analyzed.

### In vivo evaluation of the antitumor effect of Bif@DOX -NPs

A breast cancer xenograft model was established by inoculating 1.0 × 10^6^ 4T1 cells (10^7^ cells/mL) into the right leg of Balb/c mice. Once the mean tumor volume reached approximately 90 mm^3^, the mice were randomly divided into the following groups (n = 10 per group): (1) normal saline (NS), (2) Bif, (3) Bif@BSA-NPs, (4) Bif + DOX, (5) DOX-NPs and (6) Bif@DOX-NPs. The respective drugs were injected via the tail vein once every three days for a total of three times. The weight and tumor volume of mice were also monitored every other day. Three days after the last drug administration, the heart, liver, spleen, lungs, kidneys and tumor tissues were harvested and embedded in paraffin for further analysis. H&E staining was performed for histopathological analysis, and fibrosis was measured by Masson’s Trichome staining. Apoptosis was evaluated using terminal-deoxynucleotidyl transferase (TUNEL) labelling. The in-situ expression of the hypoxia inducible factor (HIF-1α) and SPARC in tumor tissues were analyzed by immunohistochemistry.

To assess early treatment response, Micro-PET/CT scans (Siemens, Germany) were performed 48 h after the last dose. Briefly, the mice were fasted at least 6 h prior to the scan, and injected with 150–250 μCi FDG. Thirty minutes later, the mice were anesthetized via isoflurane inhalation, and whole-body PET/CT scans were performed in two-dimensional mode (10 min per location emission scan) using the parameters of 80 kV, 500 mA and 1.5 mm slice collimation. The PET/CT images were analyzed by two nuclear medicine physicians. The regions of interest (ROIs) on the tumor images were drawn manually and randomly, and the maximum normalized uptake value (SUV_max_) and mean uptake value (SUV_mean_) were calculated using the hottest individual pixel within the tumor.

To investigate the in vivo drug biodistribution, Bif@DOX-NPs were injected through the tail vein, and the mice were euthanized 24 h later. The heart, liver, spleen, lungs, kidneys and tumors were harvested and homogenized, and the homogenates were centrifuged at 10,000 rpm for 10 min. The supernatant was transferred to the colorimetric cup and the fluorescence intensity was measured using a spectrophotometer (Perkin- Elmer LS-55, Smeo Analytical Instruments Co, China).

### In vitro hemolysis analysis and in vivo toxicity evaluation of bacterial nanohybrids

One milliliter erythrocyte suspension (0.2%, v/v) was mixed with 1 mL saline containing Bif, BSA-NPs or Bif@BSA-NPs, and incubated at 37 °C for 4 h. The positive control was hemolyzed in double-distilled water and saline was included as the negative control. All samples were centrifuged at 4 °C and 3000 rpm for 6 min, and the OD of the supernatant was measured at 540 nm using a UV–Vis spectrophotometer (UV-5800PC, Shanghai Metash Instruments Co., Ltd., Shanghai, China). The hemolysis rate was calculated according to the following equation

$$\mathrm{Hemolysis \, Rate }(\mathrm{\%}) = \frac{OD \, value \, of \, experimental\, group - OD \, value \, of \, saline\, group}{OD \, value \, of \, positive \, control \, group - OD \, value \, of \, saline \, group}\times 100\mathrm{\%}$$

To study the in vivo toxicity of Bif@DOX-NPs, 12 healthy SD rats were randomly divided into the NS, Bif, Bif + DOX and Bif@DOX-NPs groups (n = 3), and injected intravenously with the respective drugs every three days, three times in total. Blood was collected via the retroorbital route for measuring red blood cells (RBC), white blood cells (WBC), platelets (PLT), hemoglobin (HGB), mean hemoglobin (MCH), mean blood cell volume (MCV), alanine aminotransferase (ALT), aspartate aminotransferase (AST), creatinine (CREA), blood urea nitrogen (BUN) and creatine kinase (CK).

### Statistical analysis

All data were expressed as mean ± standard deviation (SD). Two groups were compared using the Student's t-test, and one-way analysis of variance (ANOVA) was used for multiple group comparison. Survival curves were plotted using the Kaplan–Meier method, and survival times and 95% confidence intervals were compared using the log-rank test. Statistical analyses were performed using GraphPad Prism version 6.07 (GraphPad Software, Inc). P values < 0.05 were considered statistically significant.

## Supplementary Information

**Additional file 1: Figure S1.** Average zeta potential of Bif, DOX-NPs and Bif@ DOX-NPs. **Figure S2.** In vitro analysis of binding stability between Bif and DOX-NPs. A. The photos of Bif@DOX-NPs in PBS (pH=7.4) after centrifugation at 0 and 24 hours. B. Bif@DOX-NPs solutions with (b, right) and without (a, left) addition of MMP-2. C. Dissociation rate of DOX-NPs from Bif@DOX-NPs at 0 and 24 hours. D. Dissociation rate of DOX-NPs from Bif@DOX-NPs solutions containing MMP-2 or not (Control). Results are presented as mean ± SD (n= 3). Asterisks indicate significant differences (ns: no statistical significance, ****P < 0.0001). **Figure S3.** Flow cytometric analysis of the effect of Bif on cell activity of LO2 and BEAS-2B. **Figure S4.** Flow cytometric analysis of the effect of different drugs on apoptosis of 4T1 cells. **Figure S5.** Flow cytometry analysis of apoptosis rates of Huh7 and HepG2 cells induced by co-incubation with Bif. Data are presented as mean ± SD (n= 3). Asterisks indicate significant differences (**P < 0.01). **Figure S6.** The growth of bacteria in the tumor tissue of mice injected with NS. **Figure S7.** In vivo hemolysis analysis. (A) Representative microscope photos of red blood cells after incubated with different samples. a: normal saline (NS, negative control); b: distilled water (DW, positive control); c: Bif; d: BSA-NPs; e: Bif@BSA-NPs. B. Photo of hemolysis in each group. (C) UV-vis absorption spectra of hemolysis in each group. (D) The hemolysis rate in each group. **Figure S8.** Blood count and biochemistry analysis of mice in the group of NS, Bif, Bif+DOX and Bif@DOX-NPs. Results are presented as mean ± SD (n= 3). Asterisks indicate significant differences (ns: no statistical significance, *P < 0.05, **P < 0.01, ****P < 0.0001). Abbreviations: white blood cells (WBC), red blood cells (RBC), hemoglobin (HGB), mean red blood cell haemoglobin concentration (MCHC), erythrocyte pressure volume (HCT), platelets (PLT), mean hemoglobin (MCH), blood cell volume (MCV), blood urea nitrogen (BUN), creatinine (CREA), glucose (GLU), albumin (ALB), mean alanine aminotransferase (ALT), aspartate aminotransferase (AST), Total cholesterol (TC), mean alanine aminotransferase/aspartate aminotransferase (AST/ALT), and creatine kinase (CK).

## Data Availability

All data needed to support the conclusions are present in the paper and/or the Supplementary Materials. Additional data related to this paper may be requested from the authors.
