# Supplementary material for: Bacteria-driven hypoxia targeting delivery of chemotherapeutic drug proving outcome of breast cancer
Source: J Nanobiotechnology. 2022 Apr 2;20:178. doi: 10.1186/s12951-022-01373-1 (PMC8976953; doi:10.1186/s12951-022-01373-1)
Supplement: Supplementary file 1 — Additional file 1: Figure S1. Average zeta potential of Bif, DOX-NPs and Bif@ DOX-NPs. Figure S2. In vitro analysis of binding stability between Bif and DOX-NPs. A. The photos of Bif@DOX-NPs in PBS (pH=7.4) after centrifugation at 0 and 24 hours. B. Bif@DOX-NPs solutions with (b, right) and without (a, left) addition of MMP-2. C. Dissociation rate of DOX-NPs from Bif@DOX-NPs at 0 and 24 hours. D. Dissociation rate of DOX-NPs from Bif@DOX-NPs solutions containing MMP-2 or not (Control). Results are presented as mean ± SD (n= 3). Asterisks indicate significant differences (ns: no statistical significance, ****P < 0.0001). Figure S3. Flow cytometric analysis of the effect of Bif on cell activity of LO2 and BEAS-2B. Figure S4. Flow cytometric analysis of the effect of different drugs on apoptosis of 4T1 cells. Figure S5. Flow cytometry analysis of apoptosis rates of Huh7 and HepG2 cells induced by co-incubation with Bif. Data are presented as mean ± SD (n= 3). Asterisks indicate significant differences (**P < 0.01). Figure S6. The growth of bacteria in the tumor tissue of mice injected with NS. Figure S7. In vivo hemolysis analysis. (A) Representative microscope photos of red blood cells after incubated with different samples. a: normal saline (NS, negative control); b: distilled water (DW, positive control); c: Bif; d: BSA-NPs; e: Bif@BSA-NPs. B. Photo of hemolysis in each group. (C) UV-vis absorption spectra of hemolysis in each group. (D) The hemolysis rate in each group. Figure S8. Blood count and biochemistry analysis of mice in the group of NS, Bif, Bif+DOX and Bif@DOX-NPs. Results are presented as mean ± SD (n= 3). Asterisks indicate significant differences (ns: no statistical significance, *P < 0.05, **P < 0.01, ****P < 0.0001). Abbreviations: white blood cells (WBC), red blood cells (RBC), hemoglobin (HGB), mean red blood cell haemoglobin concentration (MCHC), erythrocyte pressure volume (HCT), platelets (PLT), mean hemoglobin (MCH), blood cell volume (MCV), bl [file 12951_2022_1373_MOESM1_ESM.docx]

**Supplementary Figures**

**Bacteria-Driven Hypoxia Targeting Delivery of Chemotherapeutic Drug Proving Outcome of Breast Cancer**

Susu Xiao^a,#^, Huan Shi^a,#^, Yan Zhang^b,#^, Yu Fan^a^, Li Wang^c^, Li Xiang^a^, Yanlin Liu^a^, Ling Zhao^d^, Shaozhi Fu^a*^

*^a^Department of Oncology, the Affiliated Hospital of Southwest Medical University, Luzhou 646000, PR China*

*^b^Department of Oncology, the Affiliated TCM Hospital of Southwest Medical University, Luzhou 646000, China*

*^c^Nuclear Medicine and Molecular Imaging Key Laboratory of Sichuan Province, Luzhou 646000, China*

*^d^Department of Pharmaceutics, School of Pharmacy of Southwest Medical University, Luzhou 646000, China*


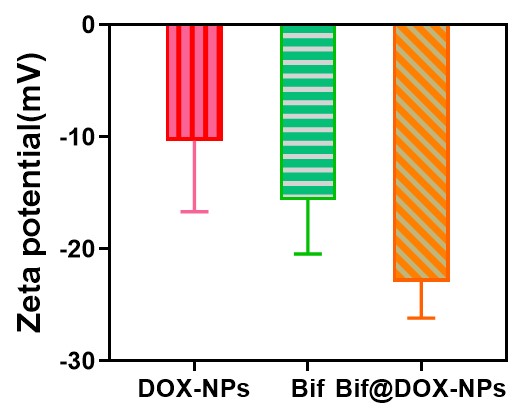


**Figure S1.** Average zeta potential of Bif, DOX-NPs and Bif@ DOX-NPs.


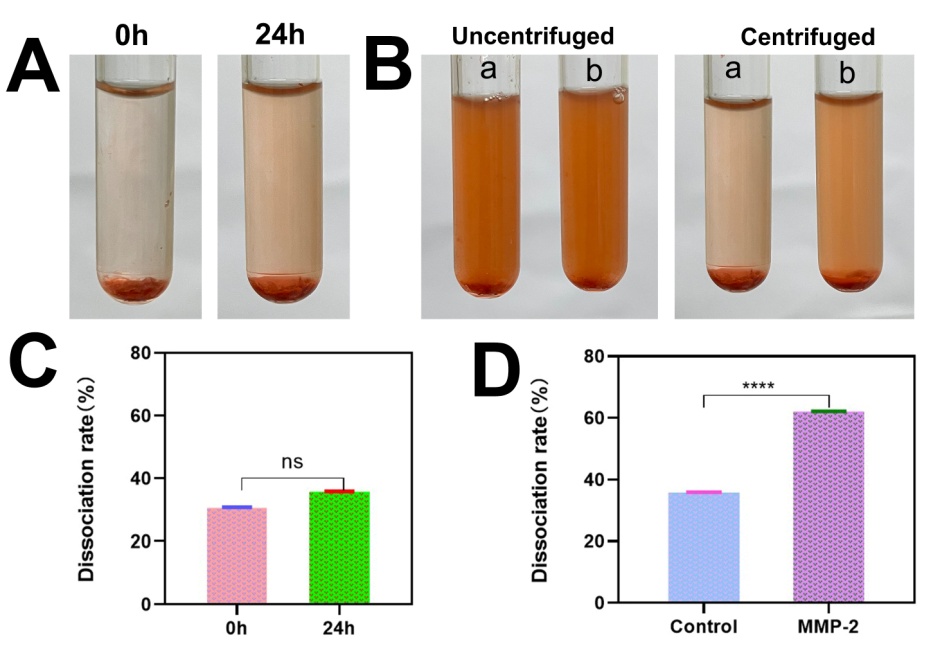


**Figure S2.** In vitro analysis of binding stability between Bif and DOX-NPs. A. The photos of Bif@DOX-NPs in PBS (pH=7.4) after centrifugation at 0 and 24 hours. B. Bif@DOX-NPs solutions with (b, right) and without (a, left) addition of MMP-2. C. Dissociation rate of DOX-NPs from Bif@DOX-NPs at 0 and 24 hours. D. Dissociation rate of DOX-NPs from Bif@DOX-NPs solutions containing MMP-2 or not (Control). Results are presented as mean ± SD (n= 3). Asterisks indicate significant differences (ns: no statistical significance, ****P < 0.0001).

**
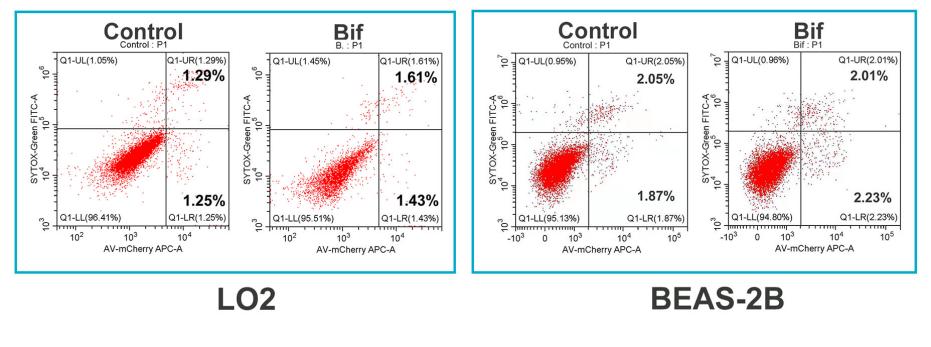
**

**Figure S3.** Flow cytometric analysis of the effect of Bif on cell activity of LO2 and BEAS-2B.

**
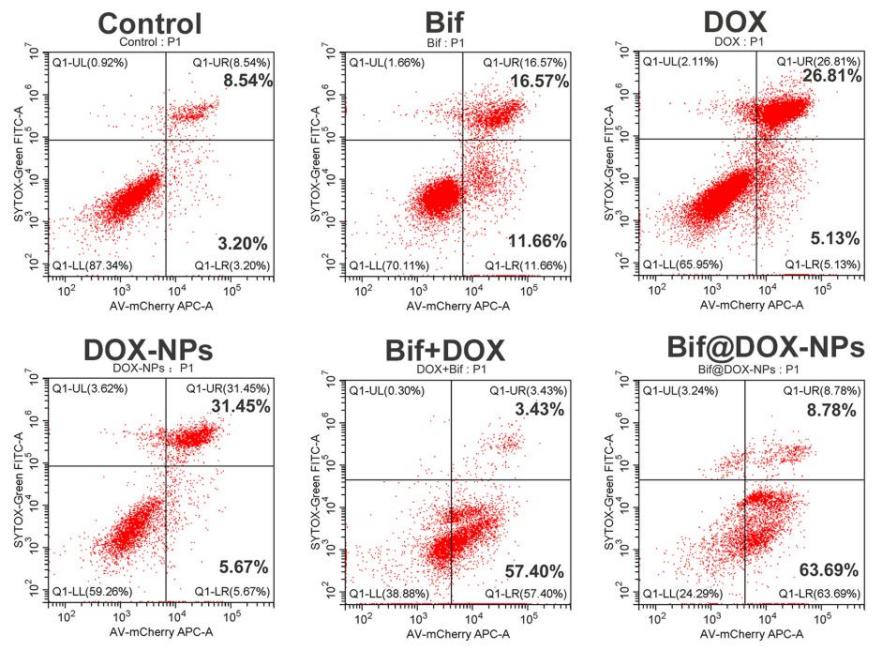
**

**Figure S4.** Flow cytometric analysis of the effect of different drugs on apoptosis of 4T1 cells.

**
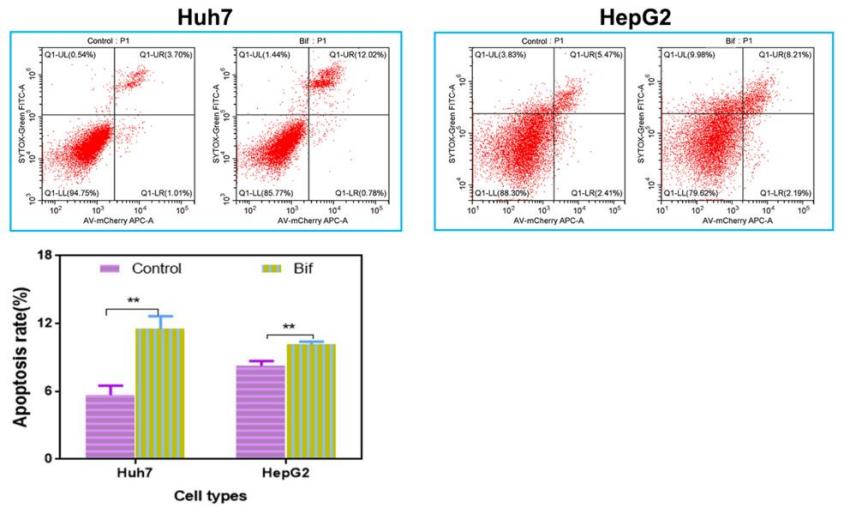
**

**Figure S5.** Flow cytometry analysis of apoptosis rates of Huh7 and HepG2 cells induced by co-incubation with Bif. Data are presented as mean ± SD (n= 3). Asterisks indicate significant differences (**P < 0.01)

**
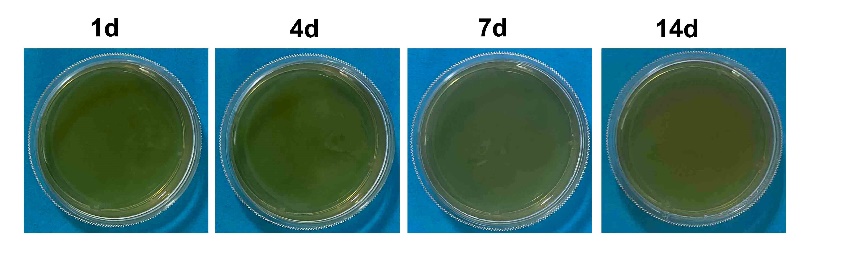
**

**Figure S6.** The growth of bacteria in the tumor tissue of mice injected with NS.

**
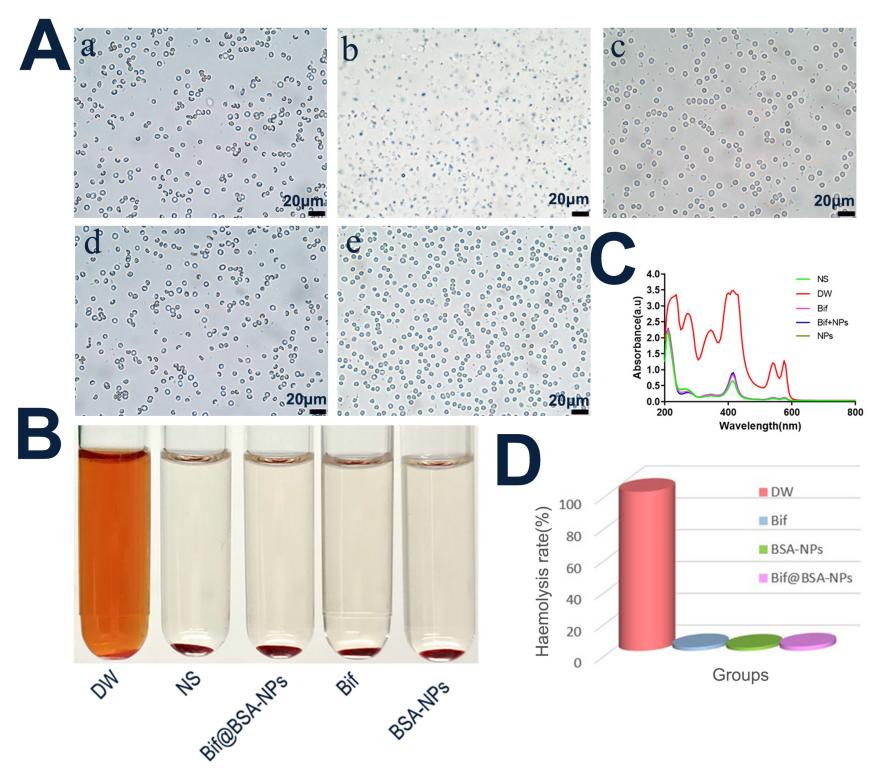
**

**Figure S7.** In vivo hemolysis analysis. (A) Representative microscope photos of red blood cells after incubated with different samples. *a: normal saline (NS, negative control); b: distilled water (DW, positive control); c: Bif; d: BSA-NPs; e: Bif@BSA-NPs.* B. Photo of hemolysis in each group. (C) UV-vis absorption spectra of hemolysis in each group. (D) The hemolysis rate in each group.


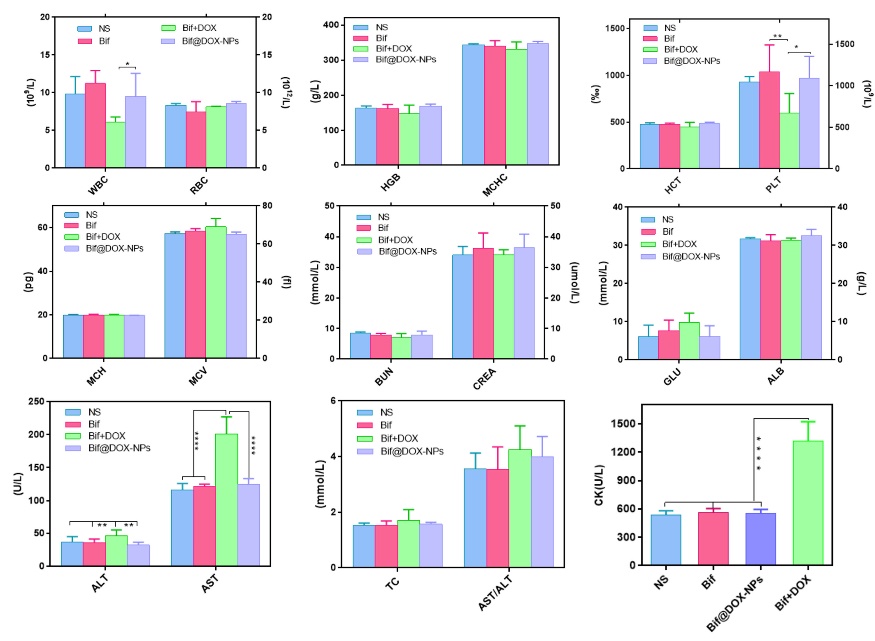


**Figure S8.** Blood count and biochemistry analysis of mice in the group of NS, Bif, Bif+DOX and Bif@DOX-NPs. Results are presented as mean ± SD (n= 3). Asterisks indicate significant differences (ns: no statistical significance, *P < 0.05, **P < 0.01, ****P < 0.0001). *Abbreviations: white blood cells (WBC), red blood cells (RBC), hemoglobin (HGB), mean red blood cell haemoglobin concentration (MCHC), erythrocyte pressure volume (HCT), platelets (PLT), mean hemoglobin (MCH), blood cell volume (MCV), blood urea nitrogen (BUN), creatinine (CREA), glucose (GLU), albumin (ALB), mean alanine aminotransferase (ALT), aspartate aminotransferase (AST), Total cholesterol (TC), mean alanine aminotransferase/aspartate aminotransferase (AST/ALT), and creatine kinase (CK).*
